# Off-label-dosing of non-vitamin K-dependent oral antagonists in AF patients before and after stroke: results of the prospective multicenter Berlin Atrial Fibrillation Registry

**DOI:** 10.1007/s00415-021-10866-2

**Published:** 2021-10-31

**Authors:** Serdar Tütüncü, Manuel Olma, Claudia Kunze, Joanna Dietzel, Johannes Schurig, Cornelia Fiessler, Carolin Malsch, Tobias Eberhard Haas, Boris Dimitrijeski, Wolfram Doehner, Georg Hagemann, Frank Hamilton, Martin Honermann, Gerhard Jan Jungehulsing, Andreas Kauert, Hans-Christian Koennecke, Bruno-Marcel Mackert, Darius Nabavi, Christian H. Nolte, Joschua Mirko Reis, Ingo Schmehl, Paul Sparenberg, Robert Stingele, Enrico Völzke, Carolin Waldschmidt, Daniel Zeise-Wehry, Peter U. Heuschmann, Matthias Endress, Karl Georg Haeusler

**Affiliations:** 1 Center for Stroke Research Berlin, Charité-Universitätsmedizin Berlin, Berlin, Germany; 2 Department of Neurology, Charité-Universitätsmedizin Berlin, Berlin, Germany; 3 Institute of Clinical Epidemiology and Biometry, University Würzburg, Würzburg, Germany; 4 Comprehensive Heart Failure Center, Clinical Trial Centre Würzburg, University of Würzburg, University Hospital Würzburg, Würzburg, Germany; 5 Department of Neurology, Vivantes Klinikum Neukölln, Berlin, Germany; 6 BCRT-Berlin Institute of Health Center for Regenerative Therapies, and Department of Cardiology (Virchow Klinikum), Charité-Universitätsmedizin Berlin, German Centre for Cardiovascular Research (DZHK), Partner Site Berlin, Berlin, Germany; 7 Department of Neurology, Helios Klinik Berlin-Buch, Berlin, Germany; 8 Department of Neurology, Vivantes Auguste-Viktoria-Klinikum, Berlin, Germany; 9 Department of Neurology, Vivantes Klinikum Spandau, Berlin, Germany; 10 Department of Neurology, Jüdisches Krankenhaus Berlin, Berlin, Germany; 11 Department of Neurology, Evangelisches Krankenhaus Königin Elisabeth Herzberge, Berlin, Germany; 12 Department of Neurology, Vivantes Klinikum im Friedrichshain, Berlin, Germany; 13 Department of Neurology, BG Klinikum Unfallkrankenhaus Berlin, Berlin, Germany; 14 Department of Neurology, German Red Cross Hospital Berlin Köpenick, Berlin, Germany; 15 Department of Neurology, Schlosspark-Klinik Berlin, Berlin, Germany; 16 Department of Neurology, Vivantes Humboldt-Klinikum, Berlin, Germany; 17 Department of Neurology, Park-Klinik Weissensee, Berlin, Germany; 18 German Center for Neurodegenerative Diseases (DZNE), Partner site Berlin, Berlin, Germany; 19 German Center for Cardiovascular Diseases (DZHK), Partner site Berlin, Berlin, Germany; 20 Berlin Institute of Health (BIH), Berlin, Germany; 21 Department of Neurology, Universitätsklinikum Würzburg, Würzburg, Germany

**Keywords:** Ischemic stroke, Atrial fibrillation, Under-dosing, NOAC

## Abstract

**Aims:**

We aimed to analyze prevalence and predictors of NOAC off-label under-dosing in AF patients before and after the index stroke.

**Methods:**

The post hoc analysis included 1080 patients of the investigator-initiated, multicenter prospective Berlin Atrial Fibrillation Registry, designed to analyze medical stroke prevention in AF patients after acute ischemic stroke.

**Results:**

At stroke onset, an off-label daily dose was prescribed in 61 (25.5%) of 239 NOAC patients with known AF and CHA_2_DS_2_-VASc score ≥ 1, of which 52 (21.8%) patients were under-dosed. Under-dosing was associated with age ≥ 80 years in patients on rivaroxaban [OR 2.90, 95% CI 1.05–7.9, *P* = 0.04; *n* = 29] or apixaban [OR 3.24, 95% CI 1.04–10.1, *P* = 0.04; *n* = 22]. At hospital discharge after the index stroke, NOAC off-label dose on admission was continued in 30 (49.2%) of 61 patients. Overall, 79 (13.7%) of 708 patients prescribed a NOAC at hospital discharge received an off-label dose, of whom 75 (10.6%) patients were under-dosed. Rivaroxaban under-dosing at discharge was associated with age ≥ 80 years [OR 3.49, 95% CI 1.24–9.84, *P* = 0.02; *n* = 19]; apixaban under-dosing with body weight ≤ 60 kg [OR 0.06, 95% CI 0.01–0.47, *P* < 0.01; *n* = 56], CHA_2_DS_2_-VASc score [OR per point 1.47, 95% CI 1.08–2.00, *P* = 0.01], and HAS-BLED score [OR per point 1.91, 95% CI 1.28–2.84, *P* < 0.01].

**Conclusion:**

At stroke onset, off-label dosing was present in one out of four, and under-dosing in one out of five NOAC patients. Under-dosing of rivaroxaban or apixaban was related to old age. In-hospital treatment after stroke reduced off-label NOAC dosing, but one out of ten NOAC patients was under-dosed at discharge.

**Clinical trial registration:**

NCT02306824.

**Supplementary Information:**

The online version contains supplementary material available at 10.1007/s00415-021-10866-2.

## Introduction

Randomized controlled trials (RCT) have demonstrated non-inferiority of non-vitamin K-dependent oral anticoagulant (NOAC) compared to the vitamin K antagonist (VKA) warfarin in (recurrent) stroke prevention in patients with non-valvular atrial fibrillation (AF) and at least moderate risk of stroke [1–4]. NOACs are superior to warfarin by reducing the risk of intracranial bleeding [5]. Present guidelines strongly recommend oral anticoagulation using a NOAC or a VKA for stroke prevention in patients with AF and prior ischemic stroke [6–8]. Registry data showed a steep increase of NOAC use for secondary stroke prevention after ischemic stroke/TIA in AF patients, even in elderly stroke patients with AF [5, 9–12]. Treating physicians are advised to prescribe the guideline-recommended in-label NOAC dosage in each patient, taking patients’ age (for dabigatran, apixaban, and edoxaban), renal function (dabigatran, apixaban, rivaroxaban, and edoxaban) as well as body weight (apixaban and edoxaban) into account [13]. According to prior investigations, up to 50% of all patients prescribed NOACs were inappropriately dosed, the vast majority of patients being under-dosed [14–17]. This is of clinical relevance, as results from a meta-analysis of randomized controlled trials as well as several registries showed that off-label NOAC dosing is associated with worse clinical outcome [18–20]. Published data on off-label dosing of NOACs is almost exclusively based on AF cohorts including a subset of patients with prior stroke. Findings on AF patients with acute ischemic stroke are rare.

The Berlin Atrial Fibrillation Registry is a multicenter prospective registry including AF patients with acute ischemic stroke/TIA. First results on factors associated with oral anticoagulation use were published previously [9]. In the present analysis, we focus on prevalence and predictors of NOAC under-dosing in AF patients before and after the index stroke.

## Methods

### Design of the registry

The design of the “Berlin Atrial Fibrillation Registry” was described in detail previously [9]. Patients with acute ischemic stroke/TIA, admitted to 1 of the 16 stroke units in Berlin, Germany, were eligible for inclusion, if patients were able to give informed consent within the in-hospital stay and either had a history of AF or a first episode of AF in-hospital. Conformation of stroke/TIA was based on brain imaging and clinical criteria, following the WHO definition [21]. No treatment recommendations were given to treating physicians at local sites.

### Data assessment

Collected information included patients’ demographics, past medical history, AF type, stroke type, individual stroke treatment, stroke severity (assessed by the National Institutes of Health Stroke Scale (NIHSS) score[22] on admission and the modified Rankin Scale (mRS) score at discharge[23]), body weight, renal function and antithrombotic medication on admission as well as at hospital discharge. The CHA_2_DS_2_-VASc and HAS-BLED score were calculated according to the current ESC guideline [6]. NOAC off-label dosing was defined according to guideline recommendations and the EHRA practical guide [6, 7, 13]. As no serum-creatinine or eGFR value was documented by the treating physicians, two patients received rivaroxaban before stroke onset and three patients after stroke were excluded from this analysis. Furthermore, two patients on apixaban at discharge were excluded because of missing values relevant to categorize regarding in- or off-label dosing (serum-creatinine level or body weight).

### Statistical methods

Statistical analysis was performed in cooperation with the Institute for Clinical Epidemiology and Biometry at the Julius-Maximilians-University of Würzburg, Germany. Baseline characteristics of study patients are presented as absolute and relative frequencies or median and quartiles. A two-sided Pearson χ2 test or Mann–Whitney *U* test was performed for univariate analyses. Analysis of predictors for the main outcomes rivaroxaban or apixaban under-dosed at stroke onset and at discharge was conducted using multiple binary logistic regression analysis and adjusted odds ratios (OR) and respective confidence intervals (CI) are reported. Parameters with *P* values < 0.05 in univariate analysis were transferred into multiple binary logistic regression analysis and variable selection was conducted using backward selection algorithm. For comparability of results, we adjusted the analysis of predictors for rivaroxaban for predictors of apixaban, and vice versa. The same strategy was applied for the multiple analyses regarding under-dosed rivaroxaban or apixaban at discharge. As sensitivity analysis, we pooled the datasets of rivaroxaban and apixaban and calculated multiple logistic regression models with outcome “rivaroxaban or apixaban” at stroke onset and at hospital discharge. In these models, variables with *P* values < 0.05 in multivariate analyses of Tables 1 and 2 or Tables 3 and 4 were included, respectively. Statistical analysis was performed using SPSS (Version 24, SPSS Inc., Chicago, USA).

**Table 1:** Characteristics of 124 registry patients with known AF and rivaroxaban prescription at stroke onset with regard to on-label dosing or off-label under-dosing

	Univariate analysis			Multivariate analysis	
	On-label dosing	Under-dosing	*P* value	OR [95% CI]*	*P* value
*n*	95	29			
Age, median [IQR]	77 [71–83]	81 [76–87]	0.02	–	–
Age ≥ 80 years, *n* (%)	35 (36.8)	18 (62.1)	0.02	2.90 [1.05–8.00]	0.04
Sex, male, *n* (%)	50 (52.6)	12 (41.4)	0.30	–	–
Body weight, median [IQR]	80 [70–90]	72 [62–82]	0.02	0.97 [0.94–1.00]	0.07
Body weight ≤ 60 kg, *n* (%)	11 (11.6)	5 (17.9)	0.39	–	–
Index event TIA, *n* (%)	36 (37.9)	7 (24.1)	0.173	–	–
Prior stroke/TIA, *n* (%)	35 (36.8)	9 (31.0)	0.60	–	–
Hypertension, *n* (%)	83 (87.4)	27 (93.1)	0.40	–	–
Congestive heart failure, *n* (%)	15 (15.8)	4 (13.0)	0.80	–	–
Diabetes, *n* (%)	32 (33.7)	11 (37.9)	0.67	–	–
Vascular disease, *n* (%)	26 (27.4)	13 (44.8)	0.08	–	–
eGFR on admission, ml/min/1.72 m^2^, median [IQR]	65 [50–80]	63 [55–71]	0.97	1.01 [0.98–1.05]	0.41
eGFR = 15–49 ml/min/1.72 m^2^, *n* (%)	25 (26.3)	0 (0.0)	< 0.01	–	–
CHA_2_DS_2_-VASc pre-stroke, median [IQR]	5.0 [3.0–6.0]	5.0 [4.0–6.0]	0.27	1.35 [0.90–2.02]	0.15
HAS-BLED pre-stroke, median [IQR]	2.0 [2.0–3.0]	2.0 [2.0–3.0]	0.43	0.56 [0.31–1.01]	0.05

*OR and 95% CI for continuous variables were expressed per point (body weight, CHA_2_DS_2_-VASc pre-stroke, HAS-BLED pre-stroke) or per unit ml/min/1.72 m^2^ (eGFR on admission)

**Table 2:** Characteristics of 61 registry patients with known AF and apixaban prescription at stroke onset with regard to on-label or off-label under-dosing

	Univariate analysis			Multivariate analysis	
	On-label dosing	Under-dosing	*P* value	OR [95% CI]*	*P* value
*n*	39	22			
Age, median [IQR]	77 [71–85]	82 [75–86]	0.16	–	–
Age ≥ 80 years, *n* (%)	17 (43.6)	15 (68.2)	0.07	3.24 [1.04–10.1]	0.04
Sex, male, *n* (%)	25 (64.1)	11 (50.0)	0.28	–	–
Body weight, median [IQR]	75 [66–87]	77 [66–83]	0.96	1.02 [0.98–1.07]	0.35
Body weight ≤ 60 kg, *n*, (%)	6 (15.4)	0 (0.0)	0.06	–	–
Index event TIA, *n* (%)	18 (46.2)	9 (40.9)	0.69	–	–
Prior stroke/TIA, *n* (%)	22 (56.4)	10 (45.5)	0.41	–	–
Hypertension, *n* (%)	35 (89.7)	21 (95.5)	0.44	–	–
Congestive heart failure, *n* (%)	6 (15.4)	4 (18.2)	0.78	–	–
Diabetes, *n* (%)	12 (30.8)	10 (45.5)	0.25	–	–
Vascular disease, *n* (%)	15 (38.5)	12 (54.5)	0.23	–	–
eGFR on admission, ml/min/1.72 m^2^, median [IQR]	66 [49–81]	52 [45–62]	0.10	0.98 [0.95–1.01]	0.20
eGFR 15–29 ml/min/1.72 m^2^, *n* (%)	4 (10.3)	0 (0.0)	0.12	–	–
Serum creatinine, ≥ 1.5 mg/dl, *n* (%)	8 (20.5)	2 (9.1)	0.25	–	–
CHA_2_DS_2_-VASc pre-stroke, median [IQR]	5 [4–6]	5 [4–6]	0.35	0.97 [0.57–1.63]	0.90
HAS-BLED pre-stroke, median [IQR]	3 [2, 3,]	3 [2–4]	0.29	1.27 [0.66–2.44]	0.48

*OR and 95% CI for continuous variables were expressed per point (body weight, CHA_2_DS_2_-VASc pre-stroke, HAS-BLED pre-stroke) or per unit ml/min/1.72 m^2^ (eGFR on admission)

**Table 3:** Characteristics of 104 registry patients with rivaroxaban at hospital discharge after the index stroke with regard to prescription of on-label or off-label under-dosing

	Univariate analysis			Multivariate analysis	
	On-label dosing	Under-dosing	*P* value	OR [95% CI]*	*P* value
*n*	85	19			
Age, median [IQR]	76 [71–81]	84 [74–88]	< 0.01	–	–
Age ≥ 80 years, *n* (%)	28 (32.9)	12 (63.2)	0.01	3.50 [1.24–9.84]	0.02
Sex, male, *n* (%)	42 (49.4)	9 (47.4)	0.87	–	–
Body weight, median [IQR]	80 [70–90]	70 [64–80]	0.04	0.98 [0.93–1.02]	0.25
Body weight < 60 kg, *n* (%)	10 (11.8)	3 (15.8)	0.63	–	–
Index event TIA, *n* (%)	34 (40.0)	6 (31.6)	0.50	–	–
NIHSS on admission, median [IQR]	2.0 [1.0–4.0]	2.0 [1.0–3.0]	0.82	–	–
NIHSS on admission, categories, *n* (%)			0.34	–	–
0–3	57 (67.1)	15 (78.9)	–	–	–
4–7	20 (23.5)	4 (21.1)	–	–	–
≥ 8	8 ( 9.4)	0 ( 0.0)	–	–	–
mRS at discharge, median [IQR]	2.0 [1.0–3.0]	2.0 [1.0–3.0]	0.59	–	–
mRS at discharge, categories, *n* (%)			0.34	–	–
0–1	35 (41.2)	5 (26.3)	–	–	–
2–3	30 (35.3)	10 (52.6)	–	–	–
4–5	20 (23.5)	4 (21.1)	–	–	–
Intravenous thrombolysis, *n* (%)	1 (1.2)	1 (5.3)	0.24	–	–
Carotid endarterectomy, *n* (%)	0 (0.0)	0 (0.0)	1.00	–	–
Endovascular thrombectomy, *n* (%)	2 (2.4)	0 (0.0)	1.00	–	–
First episode of AF in-hospital, *n* (%)	9 (10.6)	0 (0.0)	0.14	–	–
Prior stroke/TIA, *n* (%)	32 (37.6)	5 (26.3)	0.35	–	–
Hypertension, *n* (%)	72 (84.7)	17 (89.5)	0.59	–	–
Congestive heart failure, *n* (%)	15 (17.6)	5 (26.3)	0.39	1.56 [0.45–5.33]	0.49
Diabetes, *n* (%)	32 (37.6)	5 (26.3)	0.35	–	–
Vascular disease, *n* (%)	29 (34.1)	5 (26.3)	0.51	–	–
eGFR at discharge, ml/min/1.72 m^2^ median [IQR]	66 [53–78]	65 [57–74]	0.71	1.02 [0.98–1.05]	0.34
eGFR 15–49 ml/min/1.72 m^2^, *n* (%)	16 (18.8)	0 (0.0)	0.04	–	–
CHA_2_DS_2_-VASc post-stroke, median [IQR]	6.0 [5.0–7.0]	6.0 [5.0–6.0]	0.84	0.89 [0.52–1.51]	0.66
HAS-BLED post-stroke, median [IQR]	3.0 [3.0–3.0]	3.0 [3.0–3.0]	0.56	1.23 [0.53–2.87]	0.63

*OR and 95% CI for continuous variables were expressed per point (body weight, CHA_2_DS_2_-VASc post-stroke, HAS-BLED post-stroke) or per unit ml/min/1.72 m^2^ (eGFR at discharge)

**Table 4:** Characteristics of 438 registry patients with apixaban at hospital discharge after the index stroke with regard to prescription of on-label or off-label under-dosing

	Univariate analysis			Multivariate analysis	
	On-label dosing	Under-dosing	*P* value	OR [95% CI]	*P* value
*n*	382	56			
Age, median [IQR]	77 [72–82]	81 [78–86]	< 0.01	–	–
Age ≥ 80 years, *n* (%)	147 (38.5)	35 (62.5)	< 0.01	1.85 [0.98–3.49]	0.06
Sex, male, *n* (%)	203 (53.1)	28 (50.0)	0.66		
Body weight, median [IQR]	78 [67–88]	74 [68–80]	0.41		
Body weight ≤ 60 kg, *n* (%)	56 (14.7)	1 (1.8)	< 0.01	0.06 [0.01–0.47]	< 0.01
Index event TIA, *n* (%)	83 (21.7)	13 (23.2)	0.80	–	–
NIHSS on admission (median [IQR])	2.0 [1.0–5.0]	3.0 [1.0–6.0]	0.44	–	–
NIHSS on admission, (*n*) (%)			0.24	–	–
0–3	242 (63.4)	29 (51.8)	–	–	–
4–7	92 (24.1)	17 (30.4)	–	–	–
≥ 8	48 (12.6)	10 (17.9)	–	–	–
mRS at discharge (median [IQR])	2.0 [1.0–3.0]	3.0 [1.0–4.0]	0.20	–	–
mRS at discharge, categories, *n* (%)			0.21	–	–
0–1	113 (29.6)	15 (26.8)	–	–	–
2–3	186 (48.7)	23 (41.1)	–	–	–
4–5	83 (21.7)	18 (32.1)	–	–	–
Intravenous thrombolysis, *n* (%)	69 (18.1)	11 (19.6)	0.78	–	–
Carotid endarterectomy, *n* (%)	2 (0.5)	1 (1.8)	0.29	–	–
Endovascular thrombectomy, *n* (%)	33 (8.6)	4 (7.1)	0.71	–	–
First episode of AF in-hospital, *n* (%)	147 (38.5)	13 (23.1)	0.03	0.63 [0.31–1.27]	0.19
Prior stroke/TIA, *n* (%)	108 (28.3)	18 (32.1)	0.55	–	–
Hypertension, *n* (%)	339 (88.7)	52 (92.9)	0.35	–	–
Congestive heart failure, *n* (%)	39 (10.2)	12 (21.4)	0.02	1.17 [0.47–2.90]	0.74
Diabetes, *n* (%)	99 (25.9)	17 (30.4)	0.48	–	–
Vascular disease, *n* (%)	98 (25.7)	20 (35.7)	0.11	–	–
eGFR at discharge, ml/min/1.72 m^2^ median [IQR]	66 [54–81]	56 [44–69]	< 0.01	0.99 [0.97–1.01]	0.16
eGFR 15–29 ml/min/1.72 m^2^, *n* (%)	10 (2.6)	0 (0.0)	0.22	–	–
Serum creatinine ≥ 1.5 mg/dl, *n* (%)	32 (8.4)	6 (10.7)	0.56	–	–
CHA_2_DS_2_-VASc post-stroke, median [IQR]	6.0 [5.0–6.0]	6.0 [6.0–7.0]	< 0.01	1.47 [1.08–2.00]	0.01
HAS-BLED post-stroke, median [IQR]	3.0 [3.0–4.0]	4.0 [3.0–4.0]	< 0.01	1.91 [1.28–2.84]	< 0.01

*OR and 95% CI for continuous variables were expressed per point (CHA_2_DS_2_-VASc post-stroke, HAS-BLED post-stroke) or per unit ml/min/1.72 m^2^ (eGFR at discharge

## Results

**Fig. 1:**
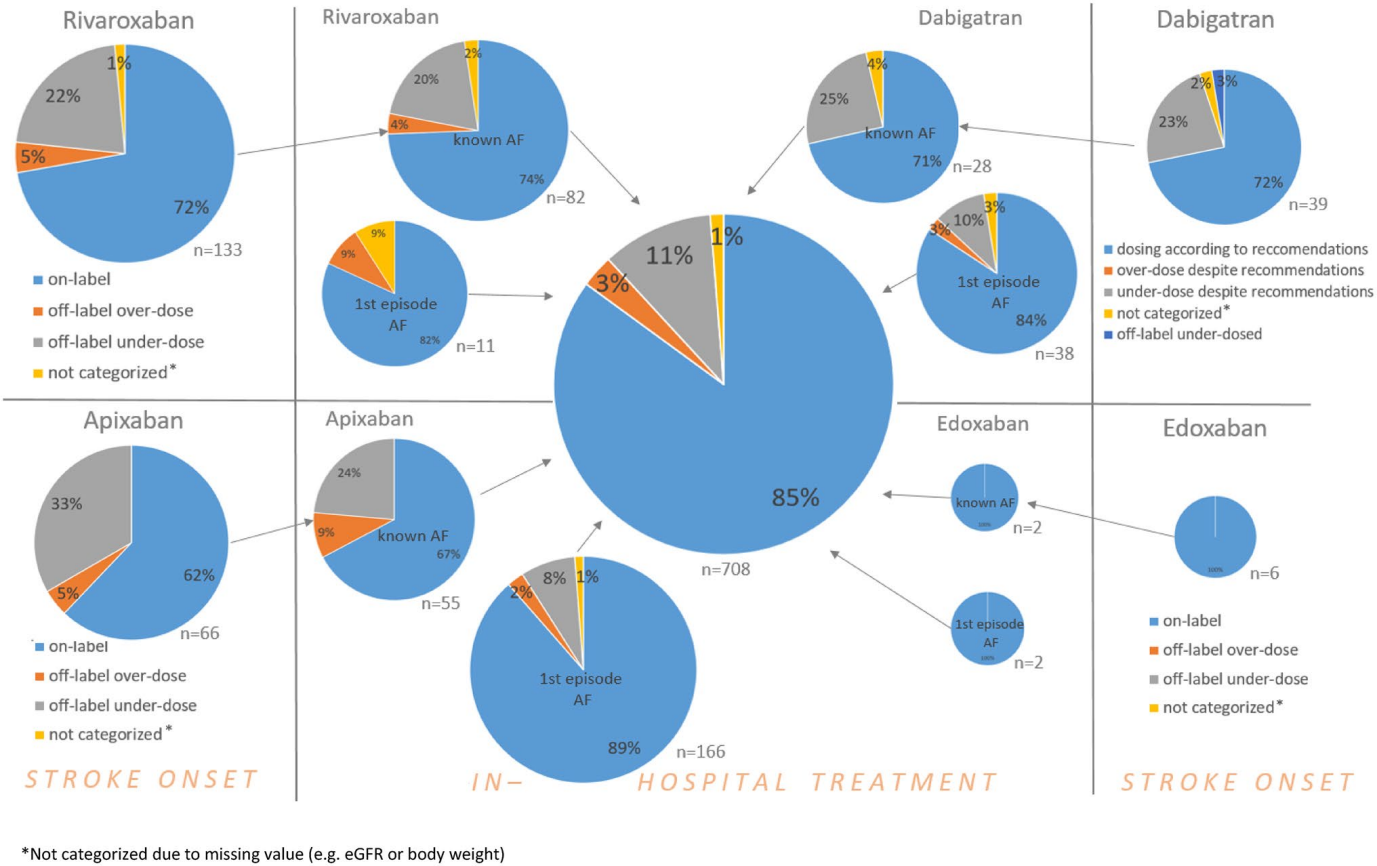
Dosing of NOACs at stroke onset and at hospital discharge in patients with known AF before stroke and in patients with a first episode of AF after acute ischemic stroke/TIA as well as in all AF patients at hospital discharge. *Not categorized due to missing value (e.g., eGFR or body weight)

Derivation of study population is shown in Fig. 1 online supplement. Baseline characteristics of the analyzed cohorts are presented in Table 1 online supplement. Of 1080 registry patients, 36 were excluded due to violation of in- or exclusion criteria, 753 (69.7%) of 1044 patients had known AF and a CHA_2_DS_2_-VASc score ≥ 1 pre-stroke. Of these 753 patients, 466 (61.9%) had been anticoagulated at stroke onset [227 (30.1%) VKA, 38 (5.0%) dabigatran, 131 (17.4%) rivaroxaban, 64 (8.5%) apixaban, and 6 (0.8%) edoxaban].

### NOAC dosing at stroke onset in patients with known AF

An off-label daily dose was prescribed in 35 (26.7%) of 131 patients on rivaroxaban, with 6 (4.5%) patients being over-dosed (receiving 20 mg OD instead of 15 mg OD), and 29 (22.1%) patients being under-dosed (receiving 10 mg OD (*n* = 1) or 15 mg OD (*n* = 28) instead of 20 mg OD). Rivaroxaban under-dosing was associated with age ≥ 80 years [OR 2.90, 95% CI 1.05–7.9, *P* = 0.04] (Table 1). An off-label daily dose of apixaban was prescribed in 25 (39.1%) of 64 patients, with 3 (4.7%) patients being over-dosed (receiving 5.0 mg TD instead of 2.5 mg TD), and 22 (34.4%) patients being under-dosed (receiving 2.5 mg TD instead of 5 mg TD). Apixaban under-dosing was associated with age ≥ 80 years [OR 3.24, 95% CI 1.04–10.1, *P* = 0.04] (Table 2). Taken together, 60 (30.8%) of 195 patients on apixaban or rivaroxaban had an off-label daily dose at stroke onset, with 51 (26.2%) patients being under-dosed. Under-dosing of rivaroxaban or apixaban was associated with age ≥ 80 years [OR 2.97, 95% CI 1.50–5.88, *P* < 0.01] (Table 2 online supplement).

Edoxaban was dosed according to label in all six patients at stroke onset. One (2.6%) out of 38 patients on dabigatran was under-dosed (receiving 75 mg TD). According to recent recommendations, an inappropriate daily dose of dabigatran was prescribed in 9 (23.7%) of 38 patients (receiving 110 mg TD instead of 150 mg TD). Prescription of dabigatran 110 mg TD vs. 150 mg TD at the time of stroke was associated with age ≥ 80 years [0% vs. 34.6%, *P* = 0.04] (Table 5 online supplement). In summary, 61 (25.5%) of 239 patients prescribed a NOAC had an off-label daily dose at stroke onset, with 52 (21.8%) patients being under-dosed.

There was not a significant impact of rivaroxaban or apixaban under-dosing vs. on-label dosing on severity of the index stroke/TIA (assessed by the NIHSS or mRS) as depicted in Table 7 Online Supplement.

### Adaption of treatment in patients with known AF and off-label NOAC dose at stroke onset

Out of six patients on rivaroxaban over-dosed at stroke onset, treating physicians switched antithrombotic medication in two patients and maintained the 20 mg OD dose in four patients (eGFR improved to ≥ 50 ml/min in one patient in-hospital). In 29 patients on rivaroxaban who were under-dosed at stroke onset, treating physicians switched antithrombotic medication in 10 patients, adapted the dose in 6 patients and maintained the 15 mg OD dose in 13 patients (eGFR declined to < 50 ml/min in 1 patient in-hospital). Subsequently, off-label rivaroxaban dosing at stroke onset was continued at hospital discharge in 15 (42.9%) out of 35 patients. In all three patients on apixaban over-dosed at stroke onset, the 5 mg TD dose was maintained. In 22 patients on apixaban under-dosed at stroke onset, treating physicians adapted the dose according to label in 10 patients and maintained the 2.5 mg TD dose in 12 patients (with creatinine remained < 1.5 mg/dl in-hospital). Subsequently, off-label apixaban dosing at stroke onset was continued at hospital discharge in 15 (60.0%) out of 25 patients. One patient receiving dabigatran 75 mg TD at stroke onset was discharged on (under-dosed) apixaban.

### NOAC dosing at hospital discharge after acute ischemic stroke or TIA

At hospital discharge, 843 (81.2%) of 1,038 registry patients with either known AF or a first episode of AF in-hospital were anticoagulated [135 (16.0%) on VKA, 134 (15.9%) on dabigatran, 111 (13.2%) on rivaroxaban, 459 (54.5%) on apixaban, and 4 (0.5%) on edoxaban], including 616 (81.3%) of 758 registry patients with known AF [126 (16.6%) on VKA, 96 (12.7%) on dabigatran, 100 (13.2%) on rivaroxaban, 292 (38.5%) on apixaban, and 2 (0.3%) on edoxaban], and 227 (79.4%) of 286 registry patients with a first episode of AF in-hospital [9 (3.1%) on VKA, 38 (13.3%) on dabigatran, 11 (3.8%) on rivaroxaban, 167 (58.4%) on apixaban, and 2 (0.7%) on edoxaban]. An off-label dose was prescribed in 97 (13.7%) of 708 NOAC patients, including 79 (11.2%) with known AF and 18 (2.5%) with a first episode of AF in-hospital (*P* < 0.01). In other terms, an off-label dose was prescribed in 79 (16.1%) of 490 with known AF and 18 (8.2%) of 218 NOAC patients with a first episode of AF in-hospital.

An off-label daily dose was prescribed in 23 (20.7%) of 111 AF patients on rivaroxaban, with 5 (4.5%) patients being over-dosed [receiving 20 mg OD instead of 15 mg OD (*n* = 4) or 30 mg OD instead of 20 mg], and 19 (17.1%) patients being under-dosed (receiving 15 mg OD instead of 20 mg OD). Rivaroxaban under-dosing at hospital discharge after stroke was associated with age ≥ 80 years [OR 3.49, 95% CI 1.24–9.84, *P* = 0.02] (Table 3).

An off-label daily dose was prescribed in 74 (16.1%) patients of 459 AF patients on apixaban, with 18 (3.9%) patients being over-dosed (receiving 5.0 mg TD instead of 2.5 mg TD), and with 56 (12.2%) patients being under-dosed (receiving 2.5 mg TD instead of 5 mg TD). Apixaban under-dosing at hospital discharge after stroke was associated with body weight ≤ 60 kg [OR 0.06, 95% CI 0.01–0.47, *P* < 0.01], CHA_2_DS_2_-VASc score [OR per point 1.47, 95% CI 1.08–2.00, *P* = 0.01], and HAS-BLED score [OR per point 1.91, 95% CI 1.28–2.84, *P* < 0.01] (Table 4).

Taken together, 97 (17.0%) of 570 patients receiving apixaban or rivaroxaban had an off-label daily dose at discharge after stroke, with 75 (13.2%) patients being under-dosed. Under-dosing of rivaroxaban or apixaban was associated with age ≥ 80 years [OR 2.27, 95% CI 1.30–3.96, *P* < 0.01], body weight ≤ 60 kg [OR 0.21, 95% CI 0.07–0.62, *P* < 0.01], a first episode of AF in-hospital [OR 0.49, 95% CI 0.25–0.95, *P* = 0.03], and the HAS-BLED score [OR per point 1.54, 95% CI 1.09–2.16, *P* = 0.01] (Table 4 online supplement).

Edoxaban was dosed on-label in all four cases prescribed after stroke. An inappropriate daily dose was prescribed in 21 (15.7%) of 134 patients on dabigatran, with 2 (1.5%) patients being over-dosed (receiving 150 mg TD instead of 110 mg TD), with 19 (14.2%) patients being under-dosed (receiving 110 mg TD instead of 150 mg TD). Prescription of dabigatran 110 mg TD instead of 150 mg TD at hospital discharge was independently associated with congestive heart failure [OR 5.15, 95% CI 1.49–18.5, *P* < 0.01] (Table 6 online supplement). Dosing of dabigatran at hospital discharge was in line with present recommendations in 108 (80.6%) out of 134 patients [24].

### NOAC off-label use

One of the dabigatran treated patients had an eGFR < 30 ml/min/1.73 m^2^ at stroke onset as well as at hospital discharge, indicating an off-label use. As none of the apixaban-, rivaroxaban- or edoxaban-treated patients had an eGFR < 15 ml/min/1.73 m^2^ on admission or at hospital discharge, there was no factor Xa inhibitor off-label use.

### Medication changes and outcome in patients on under-dosed rivaroxaban or apixaban at hospital discharge

One year after the index stroke/TIA, follow-up data on medication were available in 590 (83.3%) of 708 patients prescribed a NOAC at hospital discharge, including 54 of 75 patients discharged on under-dosed rivaroxaban or apixaban. Assuming an unchanged kidney function at hospital discharge and at 12 months, 37 (68.5%) of 54 patients prescribed under-dosed rivaroxaban or apixaban at hospital discharge were still on under-dosed rivaroxaban or apixaban, 13 (24.1%) patients were switched to the on-label dose of rivaroxaban or apixaban, 3 (5.6%) were switched to VKA or heparin in therapeutic dose, and one (1.9%) patient was switched to antiplatelet therapy at 12 months after index stroke. Further information can be found in the Online Supplement.

At 12 months after the index stroke/TIA, recurrent ischemic stroke or TIA, hemorrhagic stroke, myocardial infarction, major bleeding, systemic emboli or all-cause death occurred in 8 (14.8%) of 54 patients on under-dosed apixaban or rivaroxaban at hospital discharge, and in 54 (10.5%) of 515 patients with on-label prescription of a NOAC at hospital discharge and available follow-up information (*P* = 0.36).

## Discussion

As AF patients with acute ischemic stroke were excluded from phase III trials comparing NOAC to warfarin [25], and registries such as ORBIT AF II [14, 19], GARFIELD-AF [26], GLORIA-AF [27], SAKURA AF [28], RE-LY AF [29] and AFNET-2 Register (EORP) [30] did not focus on acute stroke patients with AF, and registries such as PRODAST (NCT02507856) or RASUNOA-prime [31] did not publish such results so far; this post hoc analysis of the prospective multicenter Berlin Atrial Fibrillation Registry is the first detailed report on NOAC off-label use in AF patients with acute ischemic stroke. We demonstrate that 26% of all registry patients with known AF prescribed a NOAC had an off-label daily dose at stroke onset, with under-dosing in the vast majority of patients (22%). Of note, apixaban was under-dosed at stroke onset in one out of three patients (33%), and rivaroxaban was under-dosed in about one out of five patients (22%). This finding is in line with a German single-center observation based on 254 acute stroke patients with known AF, reporting an off-label dose in one out of three patients on factor Xa inhibitors. [32]

In the Berlin Atrial Fibrillation Registry, under-dosing of apixaban and rivaroxaban at stroke onset was associated with old age, which is in line with prospective AF registries like ORBIT AF II, including only a subset of patients with prior (but not acute) ischemic stroke [14–17]. Compared to on-label dosing, under-dosing of apixaban or rivaroxaban was not significantly associated with stroke severity on hospital admission or at hospital discharge. Our results are not in line with previous reports, stating a lower stroke severity in unselected patients on appropriate NOAC dosing [33]. As an informed consent was requested in the Berlin Atrial Fibrillation Registry, the subsequent selection bias towards minor stroke and TIA patients has to be taken into account.

Under-dosing of rivaroxaban at hospital discharge after acute ischemic stroke or TIA was associated with old age (Table 2), while under-dosing of apixaban was associated with higher CHA_2_DS_2_-VASc as well as HAS-BLED score and inversely associated with lower body weight (Table 3). Furthermore, rivaroxaban and apixaban under-dosing at hospital discharge was associated with old age, higher HAS-BLED score and inversely associated with newly diagnosed AF in-hospital and body weight ≤ 60 kg (Table 3 online supplement). Of note, one of four patients on under-dosed daily dose of apixaban at discharge did not fulfill one of the three pre-defined dose-reduction criteria, of which two are necessary for prescribing the reduced apixaban dose according to label (Fig. 2 online supplement).

In-hospital treatment of patients with known AF and an off-label NOAC dosing at stroke onset almost halved the proportion of patients prescribed an off-label NOAC dose (26% on admission vs. 14% at discharge). However, NOAC off-label dosing was present in 14% of all registry patients (with either known AF before stroke or a first AF episode in-hospital) discharged on a NOAC (Fig. 1). As under-dosing was present in the vast majority of such patients, the fear of secondary hemorrhagic transformation and subsequent clinical worsening might have played an important role. Off-label NOAC dosing was less likely in stroke/TIA patients with a first episode of AF in-hospital and initiation of NOAC therapy than in patients with known AF before stroke (2.5% vs 11.2%, *P* < 0.01).

At 12 months after acute ischemic stroke or TIA, two-thirds of all the patients discharged on under-dosed rivaroxaban or apixaban with available follow-up information were still taking the reduced dose of apixaban or rivaroxaban. If compared to patients with on-label NOAC dosing, patients discharged on under-dosed rivaroxaban or apixaban had a similar rate of clinical endpoints (recurrent ischemic stroke or TIA, hemorrhagic stroke, myocardial infarction, major bleeding, systemic embolism or all-cause death) at 12 months after the index stroke or TIA.

Whether continued off-label dosing relates to a higher rate of recurrent stroke during the 3-year follow-up of the Berlin Atrial Fibrillation Registry remains to be analyzed [33]. Nevertheless, off-label dosing of NOACs in stroke patients should be avoided in any case, and awareness of treating physicians is needed. Whether patient identity cards including individual NOAC dose-reduction criteria or detailed documentation in obligatory quality assurance measures may be helpful to reduce off-label dosing of NOACs, has to be investigated.

As no off-label use of factor Xa inhibitors has to be reported, one patient with severe impaired kidney function was prescribed dabigatran at the time of stroke. Keeping in mind that both dabigatran dosing strategies tested in the RE-LY trial are in label in Germany, dabigatran dosing followed recent dosing recommendations in the vast majority of patients [1, 24].

The following limitations have to be addressed: first, providing informed consent by the patient implies a selection bias towards patients with less severe stroke. Second, we cannot exclude that undocumented factors have influenced the physicians’ choice of medical stroke prevention in an individual patient. Third, 0.7% of all registry patients were excluded from the present analysis due to insufficient data. Fourth, as only a few patients were on edoxaban at stroke onset, we cannot draw respective conclusions. Fifth, covering the Berlin area in total, the generalizability of the results is limited. Six, this is an exploratory analysis while the registry was not designed to analyze factors associated with NOAC off-label dosing and the impact on clinical outcome in-hospital.

## Conclusions

Off-label dosing of NOAC therapy in patients with AF is common at onset of acute stroke/TIA, and the vast majority of such patients is under-dosed. Under-dosing of rivaroxaban or apixaban was more often found in elderly AF patients. In-hospital treatment reduced the number of patients with off-label dosing by half, but one out of ten patients remained off-label under-dosed after in-hospital treatment. Compared to patients with known AF before stroke, off-label dosing was less likely in stroke patients with a first episode of AF in-hospital. Again, under-dosing of rivaroxaban or apixaban at hospital discharge was more often found in elderly AF patients and those with higher HAS-BLED scores. Further efforts are needed to reduce off-label dosing of NOACs in AF patients at risk of stroke.

## Supplementary Information

Below is the link to the electronic supplementary material.

Supplementary file1 (DOCX 204 KB)

## Data Availability

The data that support the findings of this study cannot be shared publicly due to restricted informed consent given by study patients. Reasonable requests may be directed to the corresponding author of the study.
