# Correction to: Off-label-dosing of non-vitamin K-dependent oral antagonists in AF patients before and after stroke: results of the prospective multicenter Berlin Atrial Fibrillation Registry

**DOI:** 10.1007/s00415-021-10907-w

**Published:** 2021-11-27

**Authors:** Serdar Tütüncü, Manuel Olma, Claudia Kunze, Joanna Dietzel, Johannes Schurig, Cornelia Fiessler, Carolin Malsch, Tobias Eberhard Haas, Boris Dimitrijeski, Wolfram Doehner, Georg Hagemann, Frank Hamilton, Martin Honermann, Gerhard Jan Jungehulsing, Andreas Kauert, Hans-Christian Koennecke, Bruno-Marcel Mackert, Darius Nabavi, Christian H. Nolte, Joschua Mirko Reis, Ingo Schmehl, Paul Sparenberg, Robert Stingele, Enrico Völzke, Carolin Waldschmidt, Daniel Zeise-Wehry, Peter U. Heuschmann, Matthias Endres, Karl Georg Haeusler

**Affiliations:** 1 Center for Stroke Research Berlin, Charité-Universitätsmedizin Berlin, Berlin, Germany; 2 Department of Neurology, Charité-Universitätsmedizin Berlin, Berlin, Germany; 3 Institute of Clinical Epidemiology and Biometry, University Würzburg, Würzburg, Germany; 4 Comprehensive Heart Failure Center, Clinical Trial Centre Würzburg, University of Würzburg, University Hospital Würzburg, Würzburg, Germany; 5 Department of Neurology, Vivantes Klinikum Neukölln, Berlin, Germany; 6 BCRT-Berlin Institute of Health Center for Regenerative Therapies, and Department of Cardiology (Virchow Klinikum), Charité-Universitätsmedizin Berlin, German Centre for Cardiovascular Research (DZHK), Partner Site Berlin, Berlin, Germany; 7 Department of Neurology, Helios Klinik Berlin-Buch, Berlin, Germany; 8 Department of Neurology, Vivantes Auguste-Viktoria-Klinikum, Berlin, Germany; 9 Department of Neurology, Vivantes Klinikum Spandau, Berlin, Germany; 10 Department of Neurology, Jüdisches Krankenhaus Berlin, Berlin, Germany; 11 Department of Neurology, Evangelisches Krankenhaus Königin Elisabeth Herzberge, Berlin, Germany; 12 Department of Neurology, Vivantes Klinikum im Friedrichshain, Berlin, Germany; 13 Department of Neurology, BG Klinikum Unfallkrankenhaus Berlin, Berlin, Germany; 14 Department of Neurology, German Red Cross Hospital Berlin Köpenick, Berlin, Germany; 15 Department of Neurology, Schlosspark-Klinik Berlin, Berlin, Germany; 16 Department of Neurology, Vivantes Humboldt-Klinikum, Berlin, Germany; 17 Department of Neurology, Park-Klinik Weissensee, Berlin, Germany; 18 German Center for Neurodegenerative Diseases (DZNE), Partner site Berlin, Berlin, Germany; 19 German Center for Cardiovascular Diseases (DZHK), Partner site Berlin, Berlin, Germany; 20 Berlin Institute of Health (BIH), Berlin, Germany; 21 Department of Neurology, Universitätsklinikum Würzburg, Würzburg, Germany

## Correction to: Journal of Neurology 10.1007/s00415-021-10866-2

The original version of this article unfortunately contained a mistake. Author name Matthias Endres was incorrectly written as Matthias Endress.

The correct spelling is “Matthias Endres”.

