# Supplementary material for: Off-label-dosing of non-vitamin K-dependent oral antagonists in AF patients before and after stroke: results of the prospective multicenter Berlin Atrial Fibrillation Registry
Source: J Neurol. 2021 Oct 31;269(1):470–80. doi: 10.1007/s00415-021-10866-2 (PMC8739306; doi:10.1007/s00415-021-10866-2)
Supplement: Supplementary file 1 — Supplementary file1 (DOCX 204 KB) [file 415_2021_10866_MOESM1_ESM.docx]

**ONLINE SUPPLEMENT**

**Off-label-dosing of non-vitamin K dependent oral antagonists in AF patients before and after stroke - Results of the prospective multicenter Berlin Atrial Fibrillation Registry**

Serdar Tütüncü, MD^1,2^, Manuel Olma, MD^1,2^, Claudia Kunze, BA^1^, Joanna Dietzel, MD^1^, Johannes Schurig, MD^1^, Cornelia Fiessler, PhD^3^, Carolin Malsch, M.Sc.^3,4^, Tobias Eberhard Haas^3^, Boris Dimitrijeski, MD^5^, Wolfram Doehner, MD^1,6^, Georg Hagemann, MD^7^, Frank Hamilton, MD^8^, Martin Honermann, MD^9^, Gerhard Jan Jungehulsing, MD^10^, Andreas Kauert, MD^11^*,* Hans-Christian Koennecke, MD^12^, Bruno Marcel Mackert, MD^8^, Darius Nabavi, MD^5^, Christian H. Nolte^1,2^, Joschua Mirko Reis, M.Sc.^3^, MD, Ingo Schmehl, MD^13^, Paul Sparenberg, MD^13^, Robert Stingele, MD^14^, Enrico Völzke, MD^15^, Carolin Waldschmidt, MD^16^, Daniel Zeise-Wehry, MD^17^, Peter U. Heuschmann, MD^3,4^, Matthias Endres, MD^1,2,18-20^, Karl Georg Haeusler, MD^21^

^1^ Center for Stroke Research Berlin, Charité - Universitätsmedizin Berlin, Germany

^2^  Department of Neurology, Charité – Universitätsmedizin Berlin

^3^ Institute of Clinical Epidemiology and Biometry, University Würzburg

^4^ Comprehensive Heart Failure Center, University of Würzburg, Clinical Trial Centre Würzburg, University Hospital Würzburg, Germany

^5^ Department of Neurology, Vivantes Klinikum Neukölln, Berlin, Germany

^6^ BCRT - Berlin Institute of Health Center for Regenerative Therapies, and Department of Cardiology (Virchow Klinikum), Charité- Universitätsmedizin Berlin, German Centre for Cardiovascular Research (DZHK), Partner Site Berlin, Germany

^7^ Department of Neurology, Helios Klinik Berlin-Buch, Berlin, Germany

^8^ Department of Neurology, Vivantes Auguste-Viktoria-Klinikum, Berlin, Germany

^9^ Department of Neurology, Vivantes Klinikum Spandau, Berlin, Germany

^10^ Department of Neurology, Jüdisches Krankenhaus Berlin, Germany

^11^ Department of Neurology, Evangelisches Krankenhaus Königin Elisabeth Herzberge, Berlin; Germany

^12^ Department of Neurology, Vivantes Klinikum im Friedrichshain, Berlin, Germany

^13^ Department of Neurology, BG Klinikum Unfallkrankenhaus Berlin, Germany

^14^ Department of Neurology, German Red Cross Hospital Berlin Köpenick, Germany

^15^ Department of Neurology, Schlosspark-Klinik Berlin, Germany

^16^ Department of Neurology, Vivantes Humboldt-Klinikum, Berlin; Germany

^17^ Department of Neurology, Park-Klinik Weissensee, Berlin, Germany

^18^ German Center for Neurodegenerative Diseases (DZNE), partner site Berlin, Germany

^19^ German Center for Cardiovascular Diseases (DZHK), partner site Berlin, Germany

^20^ Berlin Institute of Health (BIH), Berlin, Germany

^21^ Department of Neurology, Universitätsklinikum Würzburg, Germany

Corresponding author: Prof. Dr. Karl Georg Häusler

Department of Neurology

Universitätsklinikum Würzburg

Josef-Schneider-Str. 11

97080 Würzburg, Germany

Phone +49 931 20123755

Fax +49 931 20123488

Haeusler_K@ukw.de

**Figure 1 Online Supplement: Derivation of study population**

**Recruited (n=1,079)***

**Excluded from analysis (n=35)** due to:

- Diagnosis of TIA/Stroke revised (n=12)
- Informed consent not valid (n=4)
- Diagnosis of AF revised (n=11)
- Diagnosis of TIA/Stroke and AF revised (n=1)
- Indication of OAC other than AF (n=2)
- Former participation in Berlin AF registry (n=3)
- Lack of all data (n=2)

**Complete analysis set (n=1,044)**

Death in-hospital (n=6)

No history of AF and no OAC at stroke onset (n=578)

No oral anticoagulation at discharge (n=201)

**OAC at discharge (n=843)**

**OAC at stroke onset (n=466)**

**VKA**, n=135

**NOAC**, n=708

**Rivaroxaban**, n=111

Not categorized**, n=3

Off-label over-dosed, n=4

Off-label under-dosed, 19

**Apixaban**, n=459

Not categorized**, 2

Off-label over-dosed, n=18

Off-label under-dosed, n=56

**Edoxaban**, n=4

Off-label dosed, n=0

**Dabigatran**, n=134

Contraindicated, n=1

**VKA**, n=227

**NOAC**, n=239

**Rivaroxaban**, n=133

Not categorized**, n=2

Off-label over-dosed, n=6

Off-label under-dosed, 29

**Apixaban**, n=64

Off-label over-dosed, n=3

Off-label under-dosed, n=22

**Edoxaban**, n=6

Off-label dosed, n=0

**Dabigatran**, n=38

* Total number of recruited patients was 1,080. For one patient no informed consent was found at the study site. This patient was excluded from all following analyses. ** Not categorized due to missing value (e.g. eGFR/serum creatinine level or body weight)

**Figure 2 Online Supplement** A) Apixaban off-label lower dosing at hospital discharge (n=56). B) Apixaban on-label lower dose at discharge (n=53). Prevalence of recommended items (age≥80 years, weight ≤60 kg, creatinine level ≥ 1.5 mg/dl) to reduce the daily dose according to label (if at least two items are present).


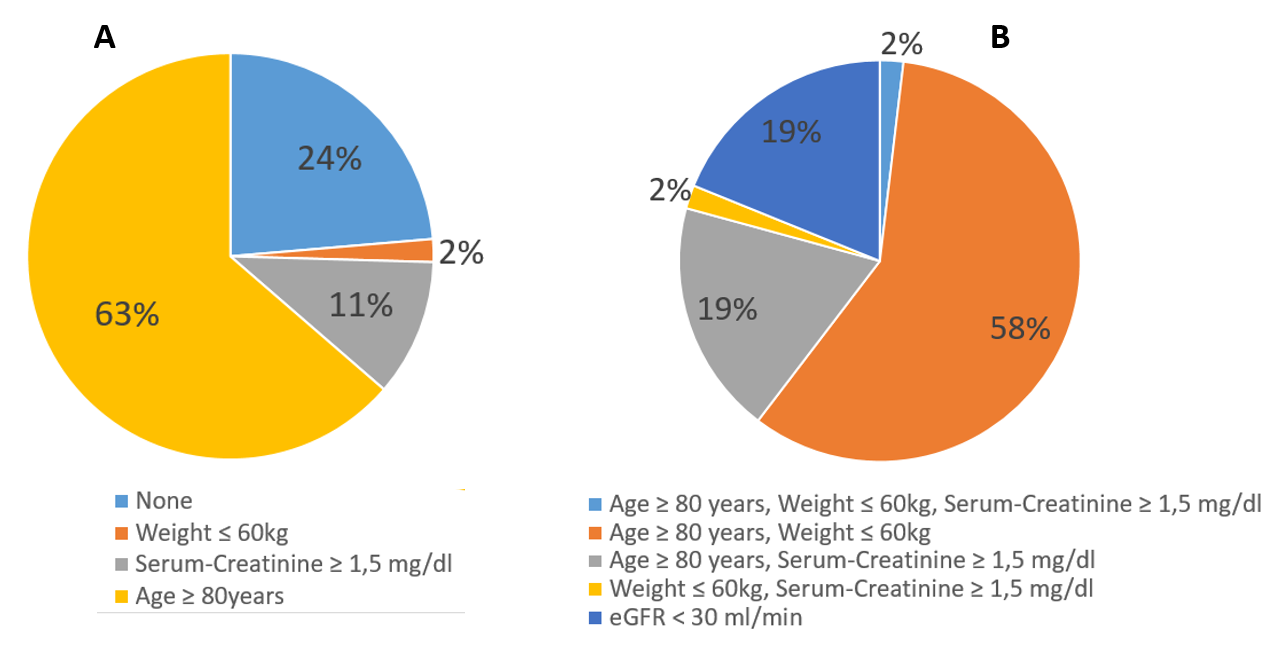


* Not categorized as on- or off-label dosing due to missing data

**Table 1 Online Supplement** Baseline characteristics of 753 registry patients with known AF before index-stroke and CHA_2_DS_2_-VASc score ≥ 1 and 1,038 survived registry patients at discharge.

|  | Registry patients with known AF before index-stroke and CHA_2_DS_2_-VASc score ≥ 1  n=753 | Surviving registry patients  at hospital discharge  n=1,038 |
| --- | --- | --- |
| Age, years (median, IQR) | 78 (72-83) | 77 (71-83) |
| Age categories, years, % (n) |  |  |
| 18-64 | 9.4 (71) | 10.6 (110) |
| 65-79 | 49.7 (375) | 50.3 (522) |
| ≥80 | 40.8 (308) | 39.1 (406) |
| Sex, female, % (n) | 48.1 (363) | 47.8 (496) |
| Index stroke, % (n) |  |  |
| Ischemic stroke | 73.1 (551) | 75.8 (787) |
| Transient ischemic attack (TIA) | 26.8 (202) | 24.2 (251) |
| NIHSS^a^ score on hospital admission, median [IQR] | 2 [1-5] | 2 [1-5] |
| modified Rankin Scale score at discharge, median [IQR] | 2 [1-3] | 2 [1-4] |
| Intravenous thrombolysis on admission, % (n) | 10.9 (82) | 15.1 (157) |
| Carotid endarterectomy, % (n) | 1.1 (8) | 1.3 (13) |
| Endovascular thrombectomy, % (n) | 6.5 (49) | 7.6 (79) |
| CHA_2_DS_2_-VASc score pre-stroke, median [IQR] | 4 [3-6] | 4 [3-5] |
| HAS-BLED score pre-stroke, median [IQR] | 3 [2-3] | 2 [2-3] |
| Cardiovascular risk factors, % (n) |  |  |
| Prior stroke or TIA | 30.9 (233) | 27.2 (282) |
| Hypertension | 89.7 (676) | 87.0 (903) |
| Heart failure | 17.7 (133) | 14.6 (152) |
| Diabetes | 31.6 (238) | 28.3 (294) |
| History of vascular disease | 32.6 (246) | 28.6 (297) |
|  |  |  |

**Table 2 Online Supplement** Multivariate analysis (backward selection) of 195 registry patients with apixaban or rivaroxaban at the time of index stroke with regard to prescription of off-label under-dosing.

|  | Multivariate Analysis | |
| --- | --- | --- |
|  | OR [95%CI] | p-value |
| Age ≥ 80 years | 2.97 [1.50-5.88] | <0.01 |
| Body weight, per point | 0.99 [0.96-1.02] | 0.43 |
| eGFR on admission, per unit mL/min/1.72m^2^ | 0.99 [0.97-1.02] | 0.70 |
| CHA_2_DS_2_-VASc pre-stroke, per point | 1.11 [0.88-1.41] | 0.37 |
| HAS-BLED pre-stroke, per point | 0.75 [0.45-1.24] | 0.26 |

**Table 3 Online Supplement**. Multivariate analysis (backward selection) of 562 patients with apixaban or rivaroxaban at discharge with regard to prescription of off-label under-dosing.

|  | Multivariate Analysis | |
| --- | --- | --- |
|  | OR [95%CI] | p-value |
| Age ≥80 years | 2.27 [1.30-3.96] | <0.01 |
| Body weight ≤60 kg | 0.21 [0.07-0.62] | <0.01 |
| First episode of AF in hospital | 0.49 [0.25-0.95] | 0.03 |
| Congestive heart failure | 1.37 [0.67-2.82] | 0.39 |
| eGFR at discharge, per unit mL/min/1.72m^2^ | 0.99 [0.98-1.01] | 0.43 |
| CHA_2_DS_2_-VASc post-stroke, per point | 1.26 [0.97-1.64] | 0.09 |
| HAS-BLED post-stroke, per point | 1.54 [1.09-2.16] | 0.01 |

## Table 4 Online Supplement Characteristics of 109 registry patients with apixaban at hospital discharge with regard to prescription of on-label lower dose or off-label under-dose.

|  | Univariate analysis | |  |
| --- | --- | --- | --- |
|  | On-label  lower dose | Off-label  lower-dose | p-value |
| n | 53 | 56 |  |
| Age, median [IQR] | 85 [81-90] | 81 [78-86] | <0.01 |
| Age ≥ 80 year, n (%) | 48 (90.6) | 35 (62.5) | <0.01 |
| Sex, % (n) = Male (%) | 15 (28.3) | 28 (50.0) | 0.02 |
| Body weight ≤60 kg, n (%) | 34 (64.2) | 1 (1.8) | < 0.01 |
| Index event TIA, n (%) | 15 (28.3) | 13 (23.2) | 0.54 |
| NIHSS on admission (median [IQR]) | 2.0 [0.0-5.0] | 3.0 [1.0-6.0] | 0.19 |
| NIHSS on admission, categories, n (%) |  |  | - |
| 0 - 3 | 37 (69.8) | 29 (51.8) | 0.08 |
| 4 - 7 | 13 (24.5) | 17 (30.4) | - |
| ≥ 8 | 3 (5.7) | 10 (17.9) | - |
| mRS at discharge (median [IQR]) | 3.0 [1.0-4.0] | 3.0 [1.0-4.0] | 0.67 |
| mRS at discharge, categories, n (%) |  |  | - |
| 0 - 1 | 17 (32.1) | 15 (26.8) | 0.82 |
| 2 - 3 | 21 (39.6) | 23 (41.1) | - |
| 4 - 5 | 15 (28.3) | 18 (32.1) | - |
| Intravenous thrombolysis, n, (%) | 5 (9.4) | 11 (19.6) | 0.13 |
| Carotid endarterectomy, n (%) | 1 (1.9) | 1 (1.8) | 0.97 |
| Endovascular treatment, n (%) | 1 (1.9) | 4 ( 7.1) | 0.19 |
| First episode of AF in hospital, n (%) | 14 (26.4) | 13 (23.2) | 0.30 |
| Prior stroke or TIA, n (%) | 17 (32.1) | 18 (32.1) | 0.99 |
| Hypertension, n (%) | 49 (92.5) | 52 (92.9) | 0.94 |
| Congestive heart failure, n (%) | 9 (17.0) | 12 (21.4) | 0.56 |
| Diabetes, n (%) | 16 (30.2) | 17 (30.4) | 0.99 |
| Vascular disease, n (%) | 22 (41.5) | 20 (35.7) | 0.53 |
| eGFR 15 - 29 , mL/min/1.72m^2^ , n (%) | 10 (18.9) | 0 (0.0) | <0.01 |
| Serum creatinine ≥ 1.5 mg/dL, n (%) | 22 (41.5) | 6 (10.7) | <0.01 |
| eGFR at discharge < 60 , mL/min/1.72m^2^ , n (%) | 39 (73.6) | 34 (60.7) | 0.15 |
| CHA_2_DS_2_-VASc post-stroke, median [IQR] | 6.0 [6.0-7.0] | 6.0 [6.0-7.0] | 0.23 |
| HAS-BLED post-stroke, median [IQR] | 4.0 [3.0-4.0] | 4.0 [3.0-4.0] | 0.42 |

## Table 5 Online Supplement Characteristics of 35* registry patients with known AF and dabigatran at stroke onset with regard to dosing according to recommendation or lower dosing despite recommendations.

|  | Univariate Analysis | |  | Multivariate Analysis | |
| --- | --- | --- | --- | --- | --- |
|  | Dose according  to recommendation | Lower dose despite recommendations | p-value | OR  [95%CI] | p-value |
| n | 26 | 9 |  |  |  |
| Age years, median [IQR] | 77 [70-83] | 75 [72-78] | 0.42 | - | - |
| Age ≥ 80 years, n (%) | 9 (34.6) | 0 (0.0) | 0.04 | n.a. | n.a. |
| Sex, male, n (%) | 14 (53.8) | 6 (66.7) | 0.50 | - | - |
| Prior stroke or TIA, n (%) | 15 (57.7) | 5 (55.6) | 0.91 | - | - |
| Hypertension, n (%) | 22 (84.6) | 7 (77.8) | 0.64 | - | - |
| Congestive heart failure, n (%) | 2 (7.7) | 2 (22.2) | 0.24 | - | - |
| Diabetes, n (%) | 12 (46.2) | 2 (22.2) | 0.21 | - | - |
| Vascular disease, n (%) | 5 (19.2) | 3 (33.3) | 0.39 | - | - |
| eGFR on admission, mL/min/1.72m^2^, median [IQR] | 68 [48-83] | 71 [60-77] | 0.93 | - | - |
| eGFR 30-50 , mL/min/1.72m^2^, n (%) | 7 (26.9) | 0 (0.0) | 0.08 | - | - |
| CHA_2_DS_2_-VASc ~~post~~pre-stroke, median [IQR] | 4 [3-6] | 5 [3-6] | 0.96 | - | - |
| CHA_2_DS_2_-VASc ~~post~~pre-stroke ≥ 4, n (%) | 20 (73.1) | 6 (66.7) | 0.71 | - | - |
| HAS-BLED ~~post~~pre-stroke, median [IQR] | 3 [2-3] | 3 [2-3] | 0.69 | - | - |
| HAS-BLED ~~post~~pre -stroke ≥ 4, n (%) | 4 (15.4) | 1 (11.1) | 0.75 | - | - |

*On admission (at the time of index stroke/TIA) 38 patients were prescribed dabigatran. Of those, three were excluded from this analysis. One patient due to off-label under-dosing with 75mg TD, one patient due to missing eGFR value, and one patient due to eGFR < 30ml/min/1.72m^2^ indicating an off-label use.

## Table 6 Online Supplement Characteristics of 127* registry patients with dabigatran at hospital discharge with regard to dosing according to recommendation or lower dosing despite given recommendations.

|  | Univariate analysis | |  | Multivariate analysis | |
| --- | --- | --- | --- | --- | --- |
|  | Dose according  to recommendations | Lower dose despite recommendations | p-  value | OR  [95%CI] | p-value |
| n | 108 | 19 |  |  |  |
| Age years, median [IQR] | 75 [67-80] | 74 [66-78] | 0.47 | - | - |
| Age ≥ 80 years, n (%) | 29 (26.9) | 0 (0) | 0.01 | n.a. | n.a. |
| Sex, male, n (%) | 58 (53.7) | 10 (52.6) | 0.93 | - | - |
| Index event TIA, n (%) | 29 (26.9) | 3 (15.8) | 0.31 | - | - |
| NIHSS, median [IQR] | 2 [1-4] | 3 [0-8] | 0.30 | - | - |
| NIHSS, categories, n (%) |  |  | 0.32 |  |  |
| 0 – 3 | 74 (68.5) | 10 (52.6) | - | - | - |
| 4 – 7 | 19 (17.6) | 4 (21.1) | - | - | - |
| ≥ 8 | 15 (13.3) | 5 (26.3) | - | - | - |
| mRS at discharge, median [IQR] | 2 [1-3] | 2 [1-3] | 0.87 | - | - |
| mRS at discharge, categories, n (%) |  |  | 0.23 | - | - |
| 0 – 1 | 38 (35.2) | 8 (42.1) | - | - | - |
| 2 – 3 | 45 (41.7) | 7 (36.8) | - | - | - |
| 4 - 5 | 25 (23.1) | 4 (21.1) | - | - | - |
| Intravenous thrombolysis, n (%) | 23 (21.3) | 4 (21.1) | 0.98 | - | - |
| Carotid endarterectomy, n (%) | 0 (0.0) | 0 (0.0) | n.a. | - | - |
| Thrombectomy, n (%) | 11 (10.2) | 0 (0.0) | 0.15 | - | - |
| First episode of AF in hospital, n (%) | 32 (29.9) | 4 (21.1) | 0.44 | - | - |
| Prior stroke or TIA, n (%) | 30 (27.8) | 6 (31.8) | 0.74 | - | - |
| Hypertension, n (%) | 79 (73.1) | 16 (84.2) | 0.31 | - | - |
| Congestive heart failure, n (%) | 7 (6.5) | 5 (26.3) | <0.01 | 5.15  [1.49-18.5] | 0.01 |
| Diabetes, n (%) | 20 (18.5) | 4 (21.1) | 0.80 | - | - |
| Vascular disease, n (%) | 23 (21.3) | 7 (36.8) | 0.14 |  |  |
| eGFR at discharge, mL/min, median [IQR] | 72 [58-82] | 71 [66-85] | 0.51 | - | - |
| eGFR 30-50mL/min, n (%) | 17 (15.9) | 0 (0.0) | 0.06 | - | - |
| CHA_2_DS_2_-VASc post-stroke, median [IQR] | 5 [4-6] | 5 [4-6] | 0.36 | - | - |
| HAS-BLED post-stroke, median [IQR] | 3 [2-3] | 3 [3-4] | 0.05 | - | - |

*At discharge 134 patients were on dabigatran. Of those, 7 were excluded from this analysis. Four due to missing eGFR value, two were off-label over-dosed, and one patient due to eGFR < 30ml/min/1.72m^2^ indicating an off-label use.

**Table 7 Online Supplement.** Impact of rivaroxaban/apixaban under-dosing and on-label dosing at stroke onset on stroke severity on admission and modified Rankin Scale (mRS) score at hospital discharge in 187 registry patients with known AF.

|  | On-label dosing  rivaroxaban  n=95 | Underdosing  rivaroxaban  n=29 | p-value |
| --- | --- | --- | --- |
| NIHSS on admission, median [IQR] | 1 [0-4] | 3 [2-4] | 0.17 |
| mRS ≤ 2  at discharge, % (n) | 67 (71) | 24 (83) | 0.19 |
|  |  |  |  |
|  | On-label dosing  apixaban  n=41 | Underdosing  apixaban  n=22 |  |
| NIHSS on admission, median [IQR] | 1 [0-4] | 2 [1-4] | 0.25 |
| mRS ≤ 2  at discharge, % (n) | 32 (78) | 14 (64) | 0.22 |
|  |  |  |  |
|  | On-label dosing  rivaroxaban or apixaban  n=136 | Underdosing  rivaroxaban or apixaban  n=51 |  |
| NIHSS on admission, median [IQR] | 1 [0-4] | 2 [1-4] | 0.08 |
| mRS ≤ 2  at discharge, % (n) | 99 (73) | 38 (75) | 0.81 |

**Medication changes in patients with rivaroxaban or apixaban under-dosing at hospital discharge**

At 12 months after the index stroke/TIA 6 (31.6%) of 19 patients with under-dosed rivaroxaban at hospital discharge were still on rivaroxaban 15mg OD, 2 (10.5%) patients were on rivaroxaban 20mg OD, 3 (15.8%) patients on apixaban 5mg TD, 1 (5.3%) patient was on VKA, and 1 (5.3%) patient received antiplatelet therapy. In six (31.6%) patients’ information was missing. Assuming an unchanged renal function after hospital discharge, 6 (31.6%) of 19 patients were still on under-dosed rivaroxaban at 12 months after the index stroke/TIA.

At 12 months after index stroke/TIA, 30 (53.7%) of 56 patients were still on apixaban 2.5mg TD, 6 (10.7%) patients were on apixaban 5mg TD, 2 (3.6%) patients were on rivaroxaban 20mg OD, one (1.8%) patient was on rivaroxaban 15mg OD, one (1.8%) patient was on VKA, and one (1.8%) patient was on heparin in therapeutic dose. In 15 (26.8%) patients information was missing (drop outs). Assuming an unchanged renal function after hospital discharge, 31 (55.4%) of 56 patients were still on under-dosed rivaroxaban or apixaban at 12 months.
